# Health professionals’ experiences and views on obstetric ultrasound in Rwanda: A cross-sectional study

**DOI:** 10.1371/journal.pone.0208387

**Published:** 2018-12-04

**Authors:** Sophia Holmlund, Joseph Ntaganira, Kristina Edvardsson, Pham Thi Lan, Jean Paul Semasaka Sengoma, Hussein Lesio Kidanto, Matilda Ngarina, Rhonda Small, Ingrid Mogren

**Affiliations:** 1 Department of Clinical Sciences, Obstetrics and Gynecology, Umeå University, Umeå, Sweden; 2 School of Public Health, College of Medicine and Health Sciences, University of Rwanda, Kigali, Rwanda; 3 Judith Lumley Centre, School of Nursing and Midwifery, La Trobe University, Melbourne, Australia; 4 Department of Dermatology and Venereology, Hanoi Medical University, Hanoi, Vietnam; 5 Department of Obstetrics and Gynecology, Muhimbili University of Health and Allied Sciences, Dar es Salaam, Tanzania; 6 Department of Obstetrics and Gynecology, Muhimbili National Hospital, Dar es Salaam, Tanzania; 7 Department of Women’s and Children’s and Reproductive Health, Karolinska Institutet, Stockholm, Sweden; Aga Khan University, KENYA

## Abstract

**Objectives:**

Implementation of ultrasound in antenatal care (ANC) in low-income countries has been shown to increase pregnant women’s compliance with ANC visits, and facilitate detection of high-risk pregnancies. In Rwanda, as in other low-income countries, access to ultrasound has increased significantly, but lack of training is often a barrier to its use. The aim of this study was to investigate Rwandan health professionals’ experiences and views of obstetric ultrasound in relation to clinical management, resources and skills.

**Methods:**

A cross-sectional questionnaire study was undertaken between November 2016 and March 2017, as part of the **CRO**ss **C**ountry **U**ltra**S**ound Study (CROCUS). Data were collected at 108 health facilities located in both rural and urban areas of Rwanda, including provincial, referral, district and private hospitals as well as health centres. Participants were obstetricians (n = 29), other physicians (n = 222), midwives (n = 269) and nurses (n = 387).

**Results:**

Obstetricians/gynecologists/other physicians commonly performed ultrasound examinations but their self-rated skill levels implied insufficient training. Access to ultrasound when needed was reported as common in hospitals, but available to a very limited extent in health centres. The vast majority of participants, independent of health profession, agreed that maternity care would improve if midwives learned to perform basic ultrasound examinations.

**Conclusions:**

Barriers to provision of high quality ultrasound services include variable access to ultrasound depending on health facility level and insufficient skills of ultrasound operators. Physicians in general need more training to perform ultrasound examinations. Implementation of a general dating ultrasound examination seems to be a relevant goal as most health professionals agree that pregnant woman would benefit from this service. To further improve maternity care services, the possibility of educating midwives to perform ultrasound examinations should be further explored.

## Introduction

Obstetric ultrasound was first introduced in the late 1950s but became more common during the 1970s and 1980s in high-income countries [1]. Nowadays, obstetric ultrasound is an established part of antenatal care (ANC) in high-resource settings. Routine ultrasound is commonly performed during the second trimester of pregnancy [2]. Although there is no consensus about whether the use of obstetric ultrasound has the ability to decrease maternal and child mortality in low-income countries [3], use of obstetric ultrasound has improved the detection of high-risk pregnancies [4, 5] and increased ANC attendance [5]. By early detection of high-risk pregnancies in ANC, pregnant women can be referred to more specialised care at higher health care levels [5]. Improved quality of dating ultrasound may also result in fewer inductions for post-term pregnancy [2]. For countries with poor access to health care, it has been shown that short courses in basic obstetric ultrasound for health professionals with no prior ultrasound experience can be effective [4]. Ultrasound training for health professionals using portable ultrasound machines has also been achieved in the most remote rural areas of Sub-Saharan Africa with promising results [6].

Rwanda is one of the poorest countries in the world but considerable effort has been invested in improving the health of the population in the last decade [7]. Rwanda has reached the Millennium Development Goal 5 with a reduction in maternal deaths from 1300/100 000 live births in 1990 to 290/100 000 live births in 2015 [8, 9]. The main reasons for the improvement in Rwanda is an increasing number of pregnant women giving birth in health facilities attended by skilled health care professionals, the introduction of a maternal death audit [10] and the use of community health workers [11]. In Rwanda, as in other low-income countries, access to obstetric ultrasound has increased significantly but lack of education and training [12, 13] as well as insufficient numbers of health professionals are often a barrier to its use [14]. Because of a lack of obstetricians/gynecologists and radiologists in Rwanda, obstetric ultrasound is largely performed by general practitioners [15]. Our earlier CROCUS study in Rwanda [13] indicated that physicians mainly learn to perform obstetric ultrasound from more experienced colleagues, and physicians sometimes have access to ultrasound machines but no skills to operate them [13]. The country report from Journal of Global Radiology, has shown that the majority of hospitals in Rwanda have ultrasound units [15], but obstetric ultrasound examinations are generally not provided at health centres where most women give birth [13, 16].

### Study rationale

Obstetric ultrasound is an important tool for pregnancy surveillance. Little is known about the access and quality of maternity ultrasound services in Rwanda. We believe that this study serves to fill a knowledge gap in the literature. The present study is part of the international **CRO**ss **C**ountry **U**ltra**S**ound Study (CROCUS) performed in three high-resource countries, one low to middle-resource country and two low-resource countries.

## Aims

The overall aim of this study was to explore different aspects of obstetric ultrasound in Rwanda from health professionals’ perspectives.

The research questions investigated were:

- What are health professionals’ views of the role of obstetric ultrasound for clinical management of pregnancy?
- How do health professionals view access to obstetric ultrasound?
- How do health professionals assess their skills in performing obstetric ultrasound examinations?
- What do health professionals believe would improve the utilisation of obstetric ultrasound?

## Materials and methods

### Study design

This study applied a cross-sectional study design with questionnaires developed for obstetricians/physicians and midwives/nurses providing pregnancy and delivery services to women in Rwanda.

### The Rwandan setting

Rwanda has five provinces: the North, East, South, and West provinces and the area of Kigali city. Every province consists of several districts [17]. The official first language is Kinyarwanda, followed by English and French [18]. The Rwandan population was estimated to be 11.8 million in 2017 [19]. The public health system has a pyramidal structure with health posts at the bottom, followed by health centres, districts hospitals and referral hospitals at the top [20]. In 2015, there were an estimated 742 physicians, 8751 nurses and 910 midwives employed in the health care system [21]. Specialised physicians represent only one fourth of the total number of physicians in the country, and they are mainly located in Kigali [22]. The number of obstetricians/gynecologists is estimated at 45 in Rwanda in 2017, according to the Rwandan Society of Obstetricians and Gynecologists (RSOG) (personal communication). There are approximately 310,000 births annually [21]. The majority of pregnant women with uncomplicated pregnancies give birth at health centres that can refer to higher levels of health care if pregnancy-related complications occur [23]. A majority (91%) of deliveries are assisted by skilled health providers, mainly nurses, partly due to the limited number of midwives and physicians [18].

### Selection of health facilities

Health facilities were selected to obtain representativeness of health professionals caring for pregnant women in Rwanda at hospital level. All provincial hospitals (n = 4) and referral hospitals (n = 7), and the largest private hospitals in Rwanda (n = 12) were included in the study [18]. In addition, four district hospitals were randomly selected in each of the four provinces and in Kigali, in total 20 district hospitals. To obtain additional experiences of health professionals at health centre level, one health centre close to every district hospital and two to three additional health centres in the rural areas of each district were also selected, totalling 108 health facilities in all. The purpose of including more health centres in rural areas than in urban areas was because the majority of health centres in Rwanda are located in rural areas.

As the data collection occurred during the rainy season it was necessary to replace three of the initially selected health facilities with three similar facilities elsewhere, due to accessibility issues.

### Sample size and power

Calculation of sample size was performed based on plausible estimations of prevalence of background and outcome variables. For the outcome requiring the largest sample size, “Every woman should undergo ultrasound examination in pregnancy to determine gestational age” and the background variable “Work experience over and under 5 years”, a sample size of 290 obstetricians/ physicians and a corresponding number of nurses/midwives (n = 290) working at hospital level, was estimated to be required to detect a difference in proportion of 0.10 with a power of 80% and a significance level of 5%. Health professionals working at health centres constituted additional participants to the number of participants estimated in the power analysis.

### Study participants

Eligible participants for the study were health professionals with different experiences of obstetric ultrasound, either working with ultrasound examinations as a major work task, *or* performing ultrasound examinations as part of their general obstetric care, *or* using the results of ultrasound in clinical management of pregnant women. Health professionals at health centres were also eligible for the study although they rarely accessed obstetric ultrasound. The primary sample consisted of 912 participants, but five participants proved to be working as radiology technicians and they were subsequently excluded from the sample. The final sample consisted of 907 participants.

### Data collection tool

The research team developed a questionnaire based on the results of the earlier qualitative studies performed in six countries in the CROCUS Study [13, 16, 24–29]. The questionnaire included items on socio-demographic characteristics, ultrasound resources and training, self-rated skills with ultrasound, and views on what may improve utilisation of ultrasound. The questionnaire included in total 105 questions and statements with fixed response options. For the focus of this publication, 42 of these items were analysed. The majority of these questions, statements and their response options are presented in Table 1.

**Table 1:** Questions and statements and their response options in the questionnaire.

*How often do you perform obstetric ultrasound examinations*? ^a^
*How do you rate your skills in ultrasound in relation to the assessment/evaluation of*: Fetal presentation^b^ Localisation of the placenta^b^ Fetal heart rate^b^ Amniotic fluid amount^b^ Gestational age estimated by CRL (crown-rump-length)^b^ Gestational age estimated by biparietal diameter, femur length and abdominal diameter^b^ Cervical length^b^ Fetal heart: 4 chamber view^b^ Fetal heart: aorta and pulmonary artery^b^ Doppler: umbilical artery^b^
*Do you have a role in decision-making regarding clinical management on the basis of obstetric ultrasound examinations*?^c^
*What do you believe would improve the utilisation of ultrasound at your clinic/work place*? More ultrasound machines^d^ Better quality of ultrasound machines^d^ More training for health professionals currently performing ultrasound^d^ More doctors trained in ultrasound^d^ (More) midwives trained in ultrasound^d^
*Statements on ultrasound resources and training* Pregnant women in my country have access to dating ultrasound (i.e. estimation of gestational age)^e^ Pregnant women in my country have access to fetal anomaly screening^e^ Pregnant women in my country have access to obstetric ultrasound independent of area of living^e^ Pregnant women in my country have access to obstetric ultrasound independent of income^e^ There are enough resources in my country to provide *medically indicated* obstetric ultrasound examinations to pregnant women who need it^e^ At my workplace, there is always access to obstetric ultrasound when it is needed^e^ At my workplace, lack of ultrasound training of the ultrasound operator sometimes leads to suboptimal pregnancy management^e^ Maternity care in my country would improve if midwives were qualified to perform basic ultrasound examinations^e^
*Statements on the role of ultrasound in clinical management of pregnancy* Ultrasound is decisive in pregnancy management^e^ Every woman should undergo ultrasound examination in pregnancy to determine gestational age^e^ It is irresponsible of a pregnant woman to decline a dating scan^e^ Ultrasound is safe to use for the pregnant woman and the fetus irrespective of the number of examinations^e^ Ultrasound is important for expectant parents to bond with their fetus during pregnancy^e^

^a^Response options: Never, On a daily basis, On a weekly basis, On a monthly basis, More seldom than on a monthly basis.

^b^Response options: No skills, Skill-level low, Skill-level intermediate, Skill-level high.

^c^Response options: No, Yes a minor role, Yes a moderate role, Yes a major role.

^d^Response options: Not at all, Not very much, A fair amount, A great deal, Don’t know.

^e^Response options: Strongly agree, Agree, Neutral, Disagree, Strongly disagree.

The questionnaire was initially developed in English and thereafter translated to French, because medical terms used in Rwandan hospitals are commonly in French. The questionnaire was pilot-tested with 10 physicians and 10 midwives/nurses at two different district hospitals in Rwanda. The participants could choose either the English or French version. Several participating midwives and nurses reported problems understanding some questions in either English or French. The questionnaire was therefore also translated into Kinyarwanda. The Kinyarwanda version was pilot-tested with five physicians and five midwives/nurses at a third district hospital. This second pilot study resulted in some minor adjustments of wording in Kinyarwanda. Parts of both the questionnaires in French and in Kinyarwanda were back-translated to English by an external person to evaluate the quality of the translation. Some wordings differed but the overall meaning was considered to be the same. Since Rwanda is a multi-lingual country, a decision was taken to provide the participants with the opportunity to choose to respond to the questionnaire in Kinyarwanda, French or English. The majority of participants chose to answer the questionnaire in Kinyarwanda, followed by French and English.

### Ethical approval

Prior to the start of the data collection, ethical approval was obtained from the University of Rwanda College of Medicine and Health Sciences Institutional Review Board, on behalf of the Rwandan National Ethics Committee (Reference No/310/CMHS IRB/2016). An approval from the Ministry of Health was also granted (Reference No 20/5779/DGPHFIS/MPP/2016).

### Data collection procedures

Two of the co-authors (JN and JPS) contacted the heads of each selected health facility to facilitate appointments for eligible participants and data collectors. Four experienced data collectors, three nurses and one clinical officer, were trained by the research team and collected all data. Data collection took place between November 2016 and March 2017. Obstetricians/physicians and midwives/nurses working on the day of the data collection at each study site were eligible participants. Data collectors distributed and collected all questionnaires at the health facilities. All participants received verbal and written information about the study and signed a consent form. Participation was voluntary. The questionnaire was completed anonymously and labelled with a unique number and a health facility code only, no identifiable data was collected. Data were entered into SPSS by two experienced data-entry clerks. To evaluate the quality of the data entry, 10% of the questionnaires including all 115 variables, were scanned and re-entered by the first author (SH). The rate of error was 0.7%. A number of items were not readable due to poor scan quality, and taking these items into account (1.3%), the error-rate theoretically varied from 0.7–2.0%. All questionnaires were stored in a secure, locked location at the University of Rwanda.

### Independent variables

The variable “current health profession” was categorised into four groups: obstetricians/gynecologists working in hospitals (OG), other physicians including general practitioners, resident physicians and radiologists working in hospitals (P), midwives/nurses working in hospitals (MNH) and nurses/midwives working in health centres (NMHC). One participant working as a medical assistant was categorised as a nurse. One OG and one P working in health centres were categorised in their respective hospital-based professional group. In some analyses obstetricians/gynecologists working in hospitals (OG) and other physicians including general practitioners, resident physicians and radiologists working in hospitals (P) were merged to a group called OGP. “Health facilities” were categorised as health centres, district hospitals and all other hospitals. District hospitals and all other hospitals were categorised as hospitals in the analysis. “Area of health facility” was categorised as health facilities in the Kigali area (n = 29) and all other health facilities outside the Kigali area (n = 79).

### Dependent variables

All dependent variables are presented in Table 1. For factors that may improve utilisation of obstetric ultrasound the dependent variables were “more ultrasound machines”, “better quality of ultrasound machines”, “more training for health professionals currently performing ultrasound”, “more physicians trained in ultrasound” and “(more) midwives trained in ultrasound” with a dichotomisation of the response options: value 0 included *not at all* and *not very much*, and value 1 included *a fair amount* and *a great deal*. For statements on ultrasound resources and training, the dependent variables were “pregnant women in my country have access to”: a) dating ultrasound (i.e. estimation of gestational age), b) fetal anomaly screening, c) obstetric ultrasound independent of area of living, d) obstetric ultrasound independent of income. For statements on ultrasound resources and training, the dependent variables were also “there are enough resources in my country to provide medically indicated obstetric ultrasound examinations to pregnant women who need it”, “at my workplace, there is always access to obstetric ultrasound when it is needed” and “at my workplace, lack of ultrasound training of the ultrasound operator sometimes leads to suboptimal pregnancy management”. The response options for the statements on ultrasound resources and training were dichotomised: value 0 included *disagree* or *strongly disagree*, and value 1 included *agree* or *strongly agree*.

### Statistics analysis

Categorical variables are presented with proportions. Continuous variables are presented with their mean values and standard deviations (SD). Pearson’s Chi-Square test was used for tests of difference of categorical data. In analysis, a p-value <0.05 was designated as statistically significant. Odds ratios (OR) and their 95% confidence intervals (CI) were calculated in univariate logistic regression analysis.

## Results

### Background characteristics of the study sample

The study sample included 907 health professionals aged 21–68 years (mean age 35.0 years). The distribution of health professionals in the total sample was obstetricians/gynecologists (3.2%), other physicians (24.5%), midwives (29.7%) and nurses (42.7%). Around 2/3 (64.2%) worked in hospitals and 35.8% in health centres (Table 2). The category “midwives/nurses at hospitals” MNH, (n = 333) consisted of a majority of midwives (70.3%) and the category “nurses/midwives at health centres” NMHC (n = 323) consisted mainly of nurses (89.2%).

**Table 2:** Background characteristics of the study sample (N = 907).

Variable^a^	All health professionals	Obstetricians/ gynecologists	Physicians, other	Midwives	Nurses
	N = 907 (100)	n = 29 (3.2)	n = 222 (24.5)	n = 269 (29.7)	n = 387 (42.7)
	n (%)	n (%)	n (%)	n (%)	n (%)
**Gender**	***907 (100)***	***29 (100)***	***222 (100)***	***269 (100)***	***387 (100)***
Male	358 (39.5)	23 (79.3)	166 (74.8)	30 (11.2)	139 (35.9)
Female	549 (60.5)	6 (20.7)	56 (25.2)	239 (88.8)	248 (64.1)
**Age, years**	***904 (99*.*7)***	***27 (93*.*1)***	***221 (99*.*5)***	***269 (100)***	***387 (100)***
Mean; SD^b^	35.0; 7.8	42.0; 9.0	32.7; 7.4	34.8; 7.4	36.1; 7.9
Min-Max	21–68	30–68	22–62	22–60	21–68
**Marital status**	***905 (99*.*8)***	***29 (100)***	***222 (100)***	***268 (99*.*6)***	***386 (99*.*7)***
Married	619 (68.2)	28 (96.6)	95 (42.8)	212 (78.8)	284 (73.4)
Cohabiting	10 (1.1)	0 (0)	0 (0)	1 (0.4)	9 (2.3)
Separated/Divorced	4 (0.4)	0 (0)	0 (0)	3 (1.1)	1 (0.3)
Widowed	19 (2.1)	0 (0)	3 (1.4)	5 (1.9)	11 (2.8)
Not married/Single	253 (27.9)	1 (3.4)	124 (55.9)	47 (17.5)	81 (20.9)
**Having children**	***894 (98*.*6)***	***29 (100)***	***219 (98*.*6)***	***264 (98*.*1)***	***382 (98*.*7)***
Yes	614 (67.7)	27 (93.1)	85 (38.3)	207 (77.0)	295 (76.2)
No	280 (30.9)	2 (6.9)	134 (60.4)	57 (21.2)	87 (22.5)
**Years in profession**	***907 (100)***	***29 (100)***	***222 (100)***	***269 (100)***	***387 (100)***
Mean; SD^b^	6.3; 6.2	8.1; 9.4	4.3; 5.4	4.5; 3.9	8.6; 6.8
Min-max	0–44	0–39	0–35	0–31	0–44
**Years in health care**	***905 (99*.*8)***	***29 (100)***	***221 (99*.*5)***	***269 (100)***	***386 (99*.*7)***
Mean; SD^b^	8.9; 7.3	12.7; 9.3	5.3; 6.0	9.2; 6.9	10.4; 7.4
Min-max	0–44	0–39	0–35	0–39	0–44
**Public/private** **health care**	***904 (99*.*7)***	***29 (100)***	***221 (99*.*5)***	***268 (99*.*6)***	***386 (99*.*7)***
Public	702 (77.4)	13 (44.8)	186 (83.8)	209 (77.7)	294 (76.0)
Private	71 (7.8)	12 (41.4)	11 (5.0)	16 (5.9)	32 (8.3)
Both public and private	131 (14.4)	4 (13.8)	24 (10.8)	43 (16.0)	60 (15.5)
**Level of health facility**	***907 (100)***	***29 (100)***	***222 (100)***	***269 (100)***	***387 (100)***
District hospital	301 (33.2)	3 (10.3)	130 (58.6)	112 (41.6)	56 (14.5)
All other hospitals^c^	281 (31.0)	25 (86.2)	91 (41.0)	122 (45.4)	43 (11.1)
Health centre	325 (35.8)	1 (3.4)	1 (0.5)	35 (13.0)	288 (74.4)
**Area of health facility**	***907 (100)***	***29 (100)***	***222 (100)***	***269 (100)***	***387 (100)***
Kigali^d^	283 (31.2)	22 (75.9)	58 (26.1)	101 (37.5)	102 (26.4)
Other areas^e^	624 (68.8)	7 (24.1)	164 (73.9)	168 (62.5)	285 (73.6)
**Provision of maternity services**^f^					
Antenatal care	647 (71.3)	28 (96.6)	166 (74.8)	176 (65.4)	277 (71.6)
Intrapartum care	775 (85.4)	27 (93.1)	200 (90.1)	254 (94.4)	294 (76.0)
Postpartum care	722 (79.6)	26 (89.7)	191 (86.0)	230 (85.5)	275 (71.1)
Do not currently provide maternity care	70 (7.7)	1 (3.4)	21 (9.5)	3 (1.1)	45 (11.6)
**Performing ultrasound**^g^	***906 (99*.*9)***	***29 (100)***	***221 (99*.*5)***	***269 (100)***	***387 (100)***
Yes	293 (32.3)	28 (96.6)	212 (95.9)	44 (16.4)	11 (2.8)
No	613 (67.6)	1 (3.4)	9 (4.1)	225 (83.6)	376 (97.2)

^a^The denominator in all calculations is the total number included in each category of health professionals.

^b^SD = Standard Deviation.

^c^Number of participants at specified health facilities included in the option “All other hospitals”: Provincial hospital (n = 58); National hospital (n = 3); Referral hospital (n = 157); Fetal medicine clinic (n = 5); Faith-based hospital (n = 2); and other type of health facility (n = 55).

^d^All levels of health facilities in the Kigali area (n = 29).

^e^All levels of health facilities in the area outside Kigali (n = 79).

^f^Item in questionnaire: “Which of the following maternity services do you provide? (Please tick all that apply)”.

^g^Performing ultrasound examinations.

### The role of obstetric ultrasound

A majority (95.9%) of all participants reported that they agreed or strongly agreed that ultrasound is decisive in pregnancy management. Most participants (79.3%-84.3%) reported that they agreed or strongly agreed that every woman should undergo ultrasound examination in pregnancy to determine gestational age. A majority (58.6%-61.6%) reported that they agreed or strongly agreed that it is irresponsible of a pregnant woman to decline a dating scan. P (90.0%), MNH (78.0%), NMHC (72.4%) and OG (65.5%) reported that they agreed or strongly agreed with the statement “Ultrasound is safe to use for the pregnant woman and the fetus irrespective of the number of examinations”. Most health professionals (79%) agreed or strongly agreed that ultrasound is important for expectant parents to bond with their fetus during pregnancy. Detailed results are presented in Table 3.

**Table 3:** The role of ultrasound in clinical management of pregnancy.

Statement^a^	Obstetricians/ gynecologists n = 29 n (%)	Physicians, other n = 222 n (%)	Midwives/ nurses at hospitals n = 333 n (%)	Nurses/ midwives at health centres n = 323 n (%)
**Ultrasound is decisive in pregnancy management**	***29 (100)***	***221 (99*.*5)***	***333 (100)***	***322 (99*.*7)***
Strongly agree	13 (44.8)	126 (56.8)	269 (80.8)	254 (78.6)
Agree	13 (44.8)	76 (34.2)	55 (16.5)	62 (19.2)
Neutral	0 (0.0)	7 (3.2)	3 (0.9)	2 (0.6)
Disagree	2 (6.9)	8 (3.6)	6 (1.8)	4 (1.2)
Strongly disagree	1 (3.4)	4 (1.8)	0 (0.0)	0 (0.0)
**Every woman should undergo ultrasound examination in pregnancy to determine gestational age**	***29 (100)***	***221 (99*.*5)***	***333 (100)***	***322 (99*.*7)***
Strongly agree	12 (41.4)	94 (42.3)	153 (45.9)	155 (48.0)
Agree	11 (37.9)	85 (38.3)	128 (38.4)	103 (31.9)
Neutral	3 (10.3)	9 (4.1)	11 (3.3)	9 (2.8)
Disagree	3 (10.3)	26 (11.7)	39 (11.7)	45 (13.9)
Strongly disagree	0 (0.0)	7 (3.2)	2 (0.6)	10 (3.1)
**It is irresponsible of a pregnant woman to decline a dating scan**	***29 (100)***	***220 (99*.*1)***	***331 (99*.*4)***	***321 (99*.*4)***
Strongly agree	10 (34.5)	69 (31.1)	102 (30.6)	117 (36.2)
Agree	7 (24.1)	60 (27.0)	92 (27.6)	81 (25.1)
Neutral	6 (20.7)	49 (22.1)	52 (15.6)	38 (11.8)
Disagree	6 (20.7)	35 (15.8)	62 (18.6)	58 (18.0)
Strongly disagree	0 (0.0)	7 (3.2)	23 (6.9)	27 (8.4)
**Ultrasound is safe to use for the pregnant woman and the fetus irrespective of the number of examinations**	***29 (100)***	***220 (99*.*1)***	***333 (100)***	***322 (99*.*7)***
Strongly agree	6 (20.7)	131 (59.0)	158 (47.4)	139 (43.0)
Agree	13 (44.8)	67 (30.2)	102 (30.6)	94 (29.1)
Neutral	6 (20.7)	15 (6.8)	46 (13.8)	44 (13.6)
Disagree	3 (10.3)	4 (1.8)	21 (6.3)	34 (10.5)
Strongly disagree	1 (3.4)	3 (1.4)	6 (1.8)	11 (3.4)
**Ultrasound is important for expectant parents to bond with their fetus during pregnancy**	***29 (100)***	***220 (99*.*1)***	***333 (100)***	***322 (99*.*7)***
Strongly agree	5 (17.2)	76 (34.2)	154 (46.2)	174 (53.9)
Agree	14 (48.3)	86 (38.7)	113 (33.9)	93 (28.8)
Neutral	6 (20.7)	41 (18.5)	27 (8.1)	28 (8.7)
Disagree	4 (13.8)	9 (4.1)	24 (7.2)	17 (5.3)
Strongly disagree	0 (0.0)	8 (3.6)	15 (4.5)	10 (3.1)

^a^The denominator in all calculations is the total number included in each category of health professionals.

### Access to obstetric ultrasound

Participants from public health facilities were less likely to report access to obstetric ultrasound when needed compared to participants from private health facilities (OR 0.16; 95% CI 0.07–0.37). Participants employed at health facilities outside the Kigali area were less likely to report access to obstetric ultrasound when needed than participants employed at health facilities in the Kigali area (OR 0.53; 95% CI 0.39–0.73). There was a significantly lower percentage of NMHC (13.2%) reporting that there is always access to ultrasound at their workplace when needed, compared with OG (93.1%), P (88.7%) and MNH (93.3%) (p<0.001; Table 4). OG were much less likely to agree that pregnant women in the country have access to obstetric ultrasound independent of area of living in comparison with NMHC (OR 0.20; 95% CI 0.08–0.46).

**Table 4:** Health professionals’ views on access to ultrasound and training (N = 907).

Statement^a^	Obstetricians/gynecologists n = 29 n (%)	Physicians, other n = 222 n (%)	Midwives/ nurses at hospitals n = 333 n (%)	Nurses/ midwives at health centres n = 323 n (%)
**Pregnant women in my country have access to dating ultrasound (i.e. estimation of gestational age)**	***29 (100)***	***222 (100)***	***332 (99*.*7)***	***321 (99*.*4)***
Strongly agree	6 (20.7)	49 (22.1)	69 (20.7)	100 (31.0)
Agree	10 (34.5)	97 (43.7)	172 (51.7)	155 (48.0)
Neutral	4 (13.8)	39 (17.6)	16 (4.8)	9 (2.8)
Disagree	6 (20.7)	30 (13.5)	64 (19.2)	45 (13.9)
Strongly disagree	3 (10.3)	7 (3.2)	11 (3.3)	12 (3.7)
**Pregnant women in my country have access to fetal anomaly screening**	***29 (100)***	***220 (99*.*1)***	***332 (99*.*7)***	***320 (99*.*1)***
Strongly agree	2 (6.9)	17 (7.7)	33 (9.9)	74 (22.9)
Agree	10 (34.5)	52 (23.4)	120 (36.0)	91 (28.2)
Neutral	4 (13.8)	59 (26.6)	53 (15.9)	47 (14.6)
Disagree	7 (24.1)	63 (28.4)	95 (28.5)	75 (23.2)
Strongly disagree	6 (20.7)	29 (13.1)	31 (9.3)	33 (10.2)
**Pregnant women in my country have access to obstetric ultrasound independent of area of living**	***29 (100)***	***221 (99*.*5)***	***331 (99*.*4)***	***321 (99*.*4)***
Strongly agree	2 (6.9)	29 (13.1)	74 (22.2)	110 (34.1)
Agree	8 (27.6)	71 (32.0)	130 (39.0)	136 (42.1)
Neutral	5 (17.2)	41 (18.5)	29 (8.7)	8 (2.5)
Disagree	8 (27.6)	54 (24.3)	78 (23.4)	44 (13.6)
Strongly disagree	6 (20.7)	26 (11.7)	20 (6.0)	23 (7.1)
**Pregnant women in my country have access to obstetric ultrasound independent of income**	***29 (100)***	***221 (99*.*5)***	***331 (99*.*4)***	***321 (99*.*4)***
Strongly agree	2 (6.9)	31 (14.0)	56 (16.8)	80 (24.8)
Agree	7 (24.1)	80 (36.0)	111 (33.3)	135 (41.8)
Neutral	5 (17.2)	35 (15.8)	32 (9.6)	21 (6.5)
Disagree	9 (31.0)	57 (25.7)	98 (29.4)	62 (19.2)
Strongly disagree	6 (20.7)	18 (8.1)	34 (10.2)	23 (7.1)
**There are enough resources in my country to provide medically indicated obstetric ultrasound examinations to pregnant women who need it**	***29 (100)***	***222 (100)***	***332 (99*.*7)***	***321 (99*.*4)***
Strongly agree	2 (6.9)	31 (14.0)	73 (21.9)	96 (29.7)
Agree	7 (24.1)	71 (32.0)	141 (42.3)	136 (42.1)
Neutral	10 (34.5)	48 (21.6)	25 (7.5)	13 (4.0)
Disagree	6 (20.7)	45 (20.3)	67 (20.1)	50 (15.5)
Strongly disagree	4 (13.8)	27 (12.2)	26 (7.8)	26 (8.0)
**At my workplace, there is always access to obstetric ultrasound when it is needed**	***29 (100)***	***222 (100)***	***330 (99*.*1)***	***317 (98*.*1)***
Strongly agree	19 (65.5)	99 (44.6)	177 (53.2)	18 (5.6)
Agree	8 (27.6)	98 (44.1)	131 (39.3)	24 (7.4)
Neutral	0 (0.0)	10 (4.5)	7 (2.1)	12 (3.7)
Disagree	1 (3.4)	9 (4.1)	12 (3.6)	93 (28.8)
Strongly disagree	1 (3.4)	6 (2.7)	3 (0.9)	170 (52.6)
**At my workplace, lack of ultrasound training of the ultrasound operator sometimes leads to suboptimal pregnancy management**	***29 (100)***	***222 (100)***	***333 (100)***	***322 (99*.*7)***
Strongly agree	2 (6.9)	50 (22.5)	114 (34.2)	103 (31.9)
Agree	14 (48.3)	82 (36.9)	126 (37.8)	98 (30.3)
Neutral	1 (3.4)	38 (17.1)	23 (6.9)	18 (5.6)
Disagree	7 (24.1)	40 (18.0)	48 (14.4)	29 (9.0)
Strongly disagree	5 (17.2)	12 (5.4)	22 (6.6)	74 (22.9)

^a^The denominator in all calculations is the total number included in each category of health professionals.

### Ultrasound operators’ self-rated skills and decision-making

**Fig 1:**
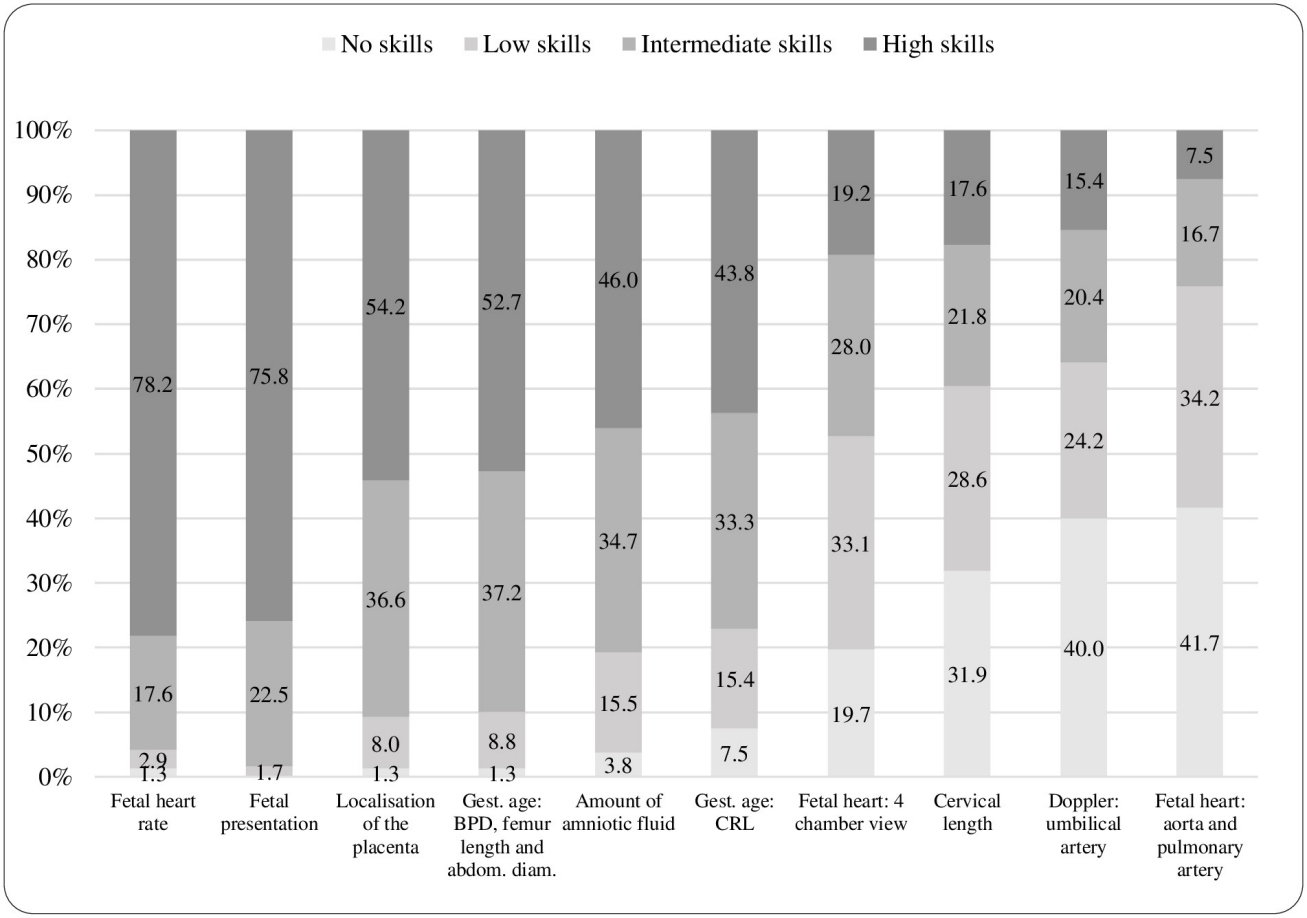
Obstetricians/Gynecologists/other physicians self-rated skills for specified ultrasound examinations (n = 240). Reported skill levels are presented with proportions (%).

**Fig 2:**
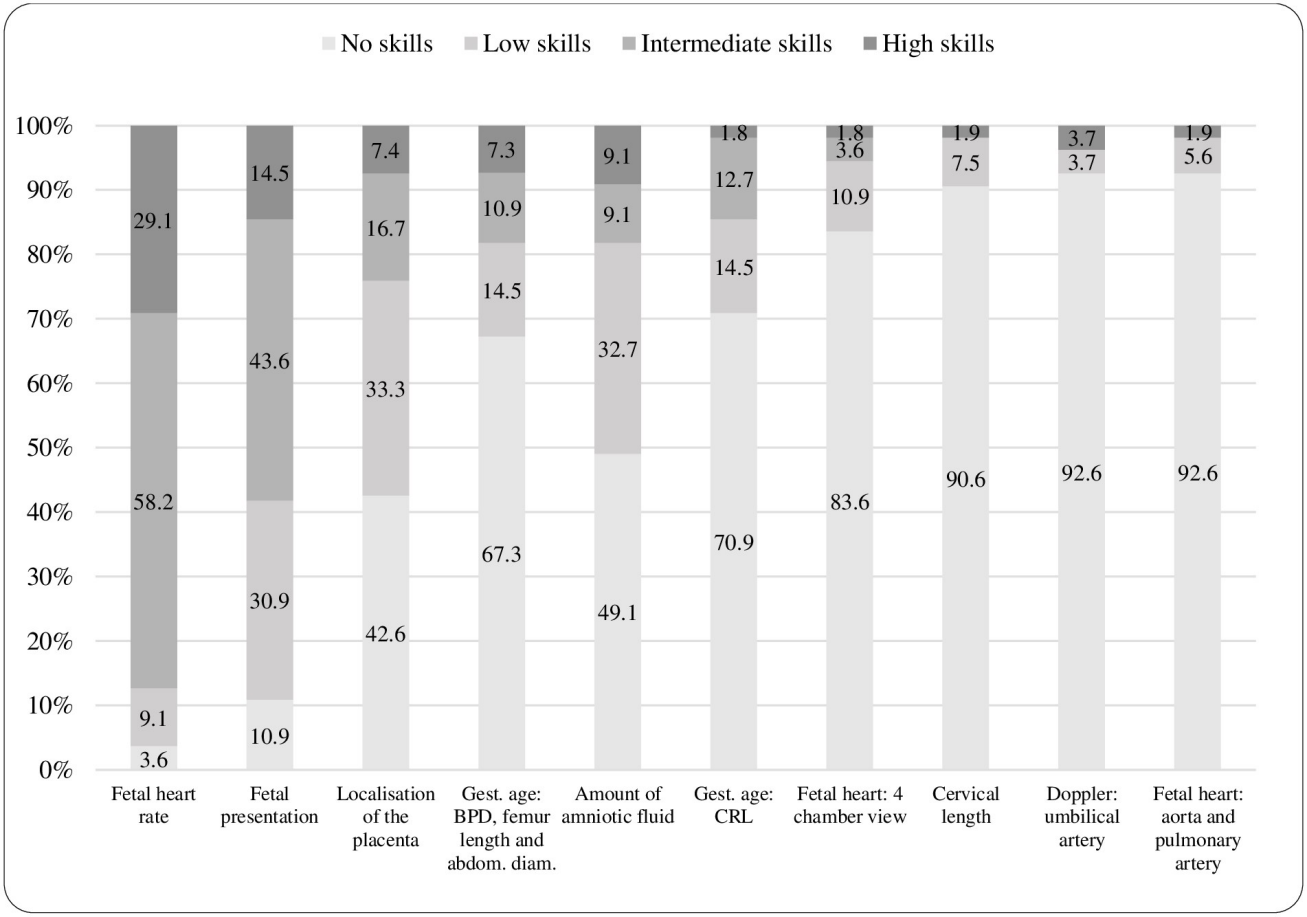
Midwives/Nurses self-rated skills for specified ultrasound examinations (n = 55). Reported skill levels are presented with proportions (%).

One third (32.3%) of the health professionals reported performing ultrasound examinations and a majority of these on a daily basis (60.1%). Almost all OG (96.6%) and P (95.9%) performed obstetric ultrasound. Small proportions of midwives (44/269; 16.4%) and nurses (11/387; 2.8%) reported performing ultrasound examinations, all working at hospitals. The following proportions of health professionals reported that midwives performed ultrasound at their workplace; NMHC (1.3%), OG (6.9%), P (13.2%) and MNH (14.7%). Obstetricians/gynecologists/other physicians (OGP) (n = 240) and midwives/nurses (MN) (n = 55) were asked to rate their skills in relation to different ultrasound examination tasks (Figs 1 and 2). Fetal heart rate was the ultrasound examination where most health professionals reported having high or intermediate skill levels (90.8%). OGP reported to a greater extent having high skills in examination of the fetal heart rate (78.2%) compared with MN (29.1%) (p<0.001). Most MN (98.2%) and OGP (75.9%) reported low or no skills for their ability to examine the fetal aorta and the fetal pulmonary artery. A majority of midwives (67.3%) stated that they had a role in decision-making regarding clinical management on the basis of obstetric ultrasound examinations while the majority of nurses (56.8%) reported they had no role in decision-making.

### Improving utilisation of obstetric ultrasound

**Fig 3:**
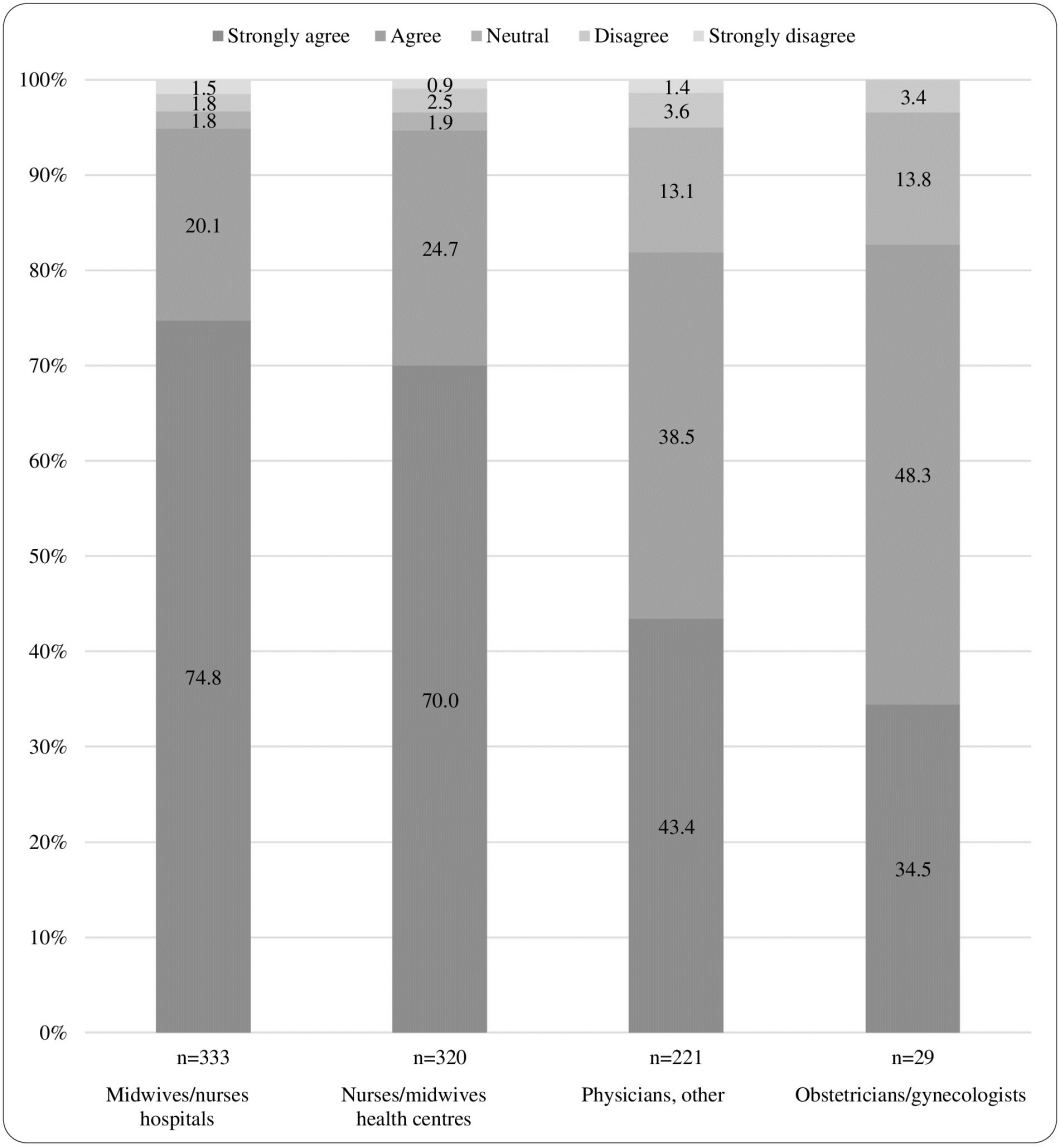
Statement from questionnaire: Maternity care in my country would improve if midwives were qualified to perform basic ultrasound examinations (N = 903). Different opinions are presented with proportions (%).

A majority of health professionals (91.3%) agreed or strongly agreed that maternity care services in Rwanda would improve if midwives were qualified to perform basic ultrasound examinations. MNH (94.9%) and NMHC (94.7%) reported most positively on the statement of midwives performing ultrasound, but also a majority of OG (82.8%) and P (81.9%) (Fig 3). NMHC were more likely to agree that more ultrasound machines would help to improve the utilisation of ultrasound in their workplace compared with OG (OR 2.96; 95% CI 1.16–7.57). Participants working in public hospitals were more likely to agree that more ultrasound machines would help to improve the utilisation of ultrasound in their workplace, compared with participants in private hospitals (OR 3.25; 95% CI 1.85–5.70). Participants not performing ultrasound were more likely to report that better quality of ultrasound machines (OR 1.79; 95% CI 1.08–2.95) and more midwives trained in ultrasound (OR 2.65; 95% CI 1.74–4.04) would help to improve the utilisation of ultrasound in their work place, compared with participants performing ultrasound. Detailed results are presented in Table 5.

**Table 5:** Health professionals’ views on factors that may improve utilisation of obstetric ultrasound.

**Variable**	**More ultrasound machines**^a^				**Better quality of ultrasound machines**^a^				
	Not at all or Not very much	A fair amount or A great deal		p-value^b^	Not at all or Not very much	A fair amount or A great deal			p-value^b^
	**n (%)**	**n (%)**			**n (%)**	**n (%)**			
**Health profession**	***134 (15*.*7)***	***719 (84*.*3)***		0.029	***68 (7*.*8)***		***806 (92*.*2)***		0.328
Obstetricians/gynecologists	7 (26.9)	19 (73.1)			3 (10.3)		26 (89.7)		
Physicians, other	38 (17.8)	176 (82.2)			23 (10.5)		197 (89.5)		
Midwives/nurses at hospitals	56 (17.8)	259 (82.2)			21 (6.6)		295 (93.4)		
Nurses/midwives at health centres	33 (11.1)	265 (88.9)			21 (6.8)		288 (93.2)		
**Public/Private health care**	***118 (16*.*3)***	***607 (83*.*7)***		<0.001	***60 (8*.*1)***		***685 (91*.*9)***		0.367
Public	96 (14.5)	567 (85.5)			52 (7.7)		624 (92.3)		
Private	22 (35.5)	40 (64.5)			8 (11.6)		61 (88.4)		
**Performing ultrasound**^c^	***134 (15*.*7)***	***718 (84*.*3)***		0.274	***68 (7*.*8)***		***805 (92*.*2)***		0.030
Yes	50 (17.9)	230 (82.1)			31 (10.8)		257 (89.2)		
No	84 (14.7)	488 (85.3)			37 (6.3)		548 (93.7)		
	**More training for health professionals currently performing ultrasound**^a^				**More physicians trained in ultrasound**^a^				
	Not at all or Not very much		A fair amount or A great deal	p-value^b^	Not at all or Not very much			A fair amount or A great deal	p-value^b^
	**n (%)**		**n (%)**		**n (%)**			**n (%)**	
**Health profession**	***48 (5*.*5)***		***828 (94*.*5)***	0.653	***60 (7*.*1)***			***789 (92*.*9)***	0.794
Obstetricians/gynecologists	2 (6.9)		27 (93.1)		1 (3.6)			27 (96.4)	
Physicians, other	14 (6.5)		203 (93.5)		18 (8.2)			201 (91.8)	
Midwives/nurses at hospitals	19 (5.9)		301 (94.1)		21 (6.9)			284 (93.1)	
Nurses/midwives at health centres	13 (4.2)		297 (95.8)		20 (6.7)			277 (93.3)	
**Public/Private health care**	***42 (5*.*6)***		***703 (94*.*4)***	0.854	***54 (7*.*5)***			***670 (92*.*5)***	1.000
Public	39 (5.8)		638 (94.2)		49 (7.4)			609 (92.6)	
Private	3 (4.4)		65 (95.6)		5 (7.6)			61 (92.4)	
**Performing ultrasound**^c^	***48 (5*.*5)***		***827 (94*.*5)***	0.242	***60 (7*.*1)***			***788 (92*.*9)***	0.495
Yes	20 (6.9)		268 (93.1)		23 (8.1)			261 (91.9)	
No	28 (4.8)		559 (95.2)		37 (6.6)			527 (93.4)	
	**(More) midwives trained in ultrasound**^a^								
	Not at all or Not very much		A fair amount or A great deal	p-value^b^					
	**n (%)**		**n (%)**						
**Health profession**	***101 (11*.*8)***		***752 (88*.*2)***	<0.001					
Obstetricians/gynecologists	4 (15.4)		22 (84.6)						
Physicians, other	45 (20.9)		170 (79.1)						
Midwives/nurses at hospitals	33 (10.5)		281 (89.5)						
Nurses/midwives at health centres	19 (6.4)		279 (93.6)						
**Public/Private health care**	***90 (12*.*4)***		***637 (87*.*6)***	0.602					
Public	80 (12.1)		581 (87.9)						
Private	10 (15.2)		56 (84.8)						
**Performing ultrasound**^c^	***101 (11*.*9)***		***751 (88*.*1)***	<0.001					
Yes	54 (19.2)		227 (80.8)						
No	47 (8.2)		524 (91.8)						

^a^Item in questionnaire: “What do you believe would improve the utilisation of ultrasound at your clinic/work place?”.

^b^Pearson’s Chi-Square test for categorical variables.

^c^Performing ultrasound examinations.

## Discussion

The main findings of this study demonstrate that obstetricians/gynecologists/other physicians commonly performed ultrasound examinations, however their self-rated skill levels in obstetric ultrasound indicate the need for further ultrasound training. Most participants agreed that ultrasound is decisive in pregnancy management. Health professionals working in hospitals commonly reported having access to ultrasound when needed, while those working in health centre reported only having very limited access. A vast majority of the participants agreed that maternity care services would improve if midwives learned to perform basic ultrasound examinations.

### Access to ultrasound

At the beginning of the 21^st^ Century the World Health Organization (WHO) recommended four ANC visits for healthy pregnant women without pregnancy-related complications [30]. In November 2016, WHO changed their recommendations from four to eight ANC contacts to improve the quality of care and to reduce maternal and perinatal mortality for all populations [31]. The new recommendations also include one ultrasound scan before 24 weeks of gestation [31]. Currently 46% of pregnant women in Rwanda receive the earlier recommended four ANC visits [32] but the estimated number of ultrasound examinations that pregnant women receive is not reported in official national data. To improve ANC attendance, introduction of obstetric ultrasound at the lowest level of care has been shown to improve the number of ANC visits and the numbers of deliveries in health facilities [33, 34]. This study found that most nurses/midwives working in health centres where most Rwandan women give birth, reported poor access to ultrasound when needed, compared with participants working in hospitals. If complications occur during pregnancy and delivery, women are referred to a higher health facility level [4]. Delay in timely treatment for obstetric complications is associated with maternal deaths and maternal near misses [35, 36]. If obstetric ultrasound could be increasingly offered at health centre level, more pregnant women would have the chance of timely referral to more qualified obstetric care. Compared with obstetricians/gynecologists in hospitals, nurses/midwives in health centres, where obstetric ultrasound is in fact rarely undertaken, were more likely to agree that pregnant women in Rwanda had access to dating ultrasound, and had access to obstetric ultrasound independent of income and area of living. A plausible explanation of this unexpected finding may be that obstetricians/gynecologists who deliver ultrasound services are aware that most pregnant women do not in fact undergo obstetric ultrasound examinations.

### Education and training of health professionals

Sub-optimal pregnancy management, because of lack of ultrasound training of operators was reported by the majority of health professionals. Ultrasound operators’ self-rated skills also indicate lack of ultrasound training. Intensive point-of-care ultrasound training with a cohort of Rwandan physicians showed that only a few obstetric ultrasound operators had any earlier structured ultrasound education [37]. Like other countries in sub-Saharan Africa, Rwanda is much affected by the limited number of health professionals and the insufficient quality of health services [38, 39]. In 2012 the Human Resources for Health (HRH) program was initiated where faculty from United States’ teaching institutions are collaborating with Rwandan Faculty to transfer skills in health care, as well as increasing the number of health professionals in Rwanda [40]. After seven years of the HRH program, Rwanda is assumed to have improved their health workforce to a sustainable level without foreign aid [41]. One of the targets identified by the HRH program was building nursing and midwifery capacity in education, leadership, research, and scholarship [42]. Midwifery education in Rwanda does now meet the International Confederation of Midwives (ICM) minimum standard of 3 years for direct-entry midwifery education or 18 months for post-graduate training [43, 44]. Through the Rwanda HRH program, the Faculty of Medicine will increase the level of skills of all physicians and broaden the workforce, including education of more specialists. The program focuses for example on strengthening the curriculum and clinical training in obstetrics and gynecology as well as radiology [40, 41]. Currently, obstetric ultrasound is mainly taught during residency programs in Rwanda, using the “Obstetric and gynecological ultrasound curriculum and competency assessment in residency training program” developed by the American Institute of Ultrasound in Medicine [45]. The HRH program has been a useful instrument to develop the health professional workforce, however, there is still a need for further improvement, especially in rural areas where the shortage of highly skilled health professionals is most pronounced [22].

The majority of participants in our study believed that ultrasound is safe to use for the pregnant woman and the fetus irrespective of the number of examinations. Taking into account the ALARA principle (As Low As Reasonably Achievable), obstetric ultrasound should only be used for medical indications and exposure should be kept as low as possible to decrease the risk for potential tissue heating and fetal compromise [46, 47].

### Improved ultrasound services

In our study, a few midwives and nurses were performing ultrasound examinations but a majority of all categories of health professionals reported the potential for improving maternity care services if midwives were allowed to become educated in performing basic ultrasound examinations. The International Society of Ultrasound in Obstetrics and Gynecology (ISUOG) recommendations for basic ultrasound examinations includes the following content: fetal viability and fetal movements, detection of multiple pregnancy, assessment of gestational age and fetal size by recording biometric measurements, evaluation of amount of amniotic fluid, placenta location and fetal position [48]. Education of midwives to perform basic ultrasound examinations would need to be sanctioned as an innovation in the Rwandan health care system. If this were to happen, access to ultrasound services at health centre level would likely increase. Although WHO recommends a minimum of 3–6 months of training with participation in 300–500 examinations for the general ultrasonography curriculum [49], several studies conclude that health professionals, mainly midwives, can be trained to perform point-of-care ultrasound to identify high-risk pregnancies [5, 50, 51] and that this can represent a low-cost improvement strategy in maternity services [52]. While lack of training is the primary barrier to use of obstetric ultrasound, maintenance and costs of machines are also important aspects [12]. In our study, the requirement for more ultrasound machines was much more likely to be reported by health professionals in public health facilities compared with those in private health care, and also for nurses/midwives in health centres compared with obstetricians/gynecologists working mainly at the highest health care levels.

## Methodological considerations

In this study, we aimed to obtain a representative sample of health professionals currently working in hospitals, but also to include health professionals working in health centres to collect additional experiences. All provincial and referral hospitals were included in the study, and 57% of all district hospitals in Rwanda. For obstetricians/gynecologists, approximately 64% of the total number working in Rwanda were included in the sample, as well as approximately 32% of other physicians and 30% of midwives in Rwanda [21]. Our assessment is that the hospital participants most probably constitute a representative sample for health professionals in hospitals in Rwanda. In addition, we consider the category of participants working in health centres to be representative for nurses/midwives at health centre level. However, taking into account all health professionals in the country managing pregnant women, mostly at health centres, our total sample cannot be considered fully representative for the whole of Rwanda. Two hundred and fifty-one obstetricians/physicians participated in the study, somewhat short of our sample size target of 290, but still one third of all physicians in Rwanda. The questionnaire was developed in English and translated into French and Kinyarwanda, and to avoid linguistic pitfalls the questionnaires were back-translated into English for quality control purposes. Although several checks were made, it is possible that some questions were not understood in exactly identical ways in the different languages. Another limitation may be the length of the questionnaire with 105 different items in total, however, the response was close to 100% for all items.

## Conclusions

In Rwanda, physicians perform most obstetric ultrasound examinations and health professionals believe that ultrasound is decisive in pregnancy management. Barriers to provision of high quality ultrasound services to pregnant women include variable access depending on health facility level and insufficient skills of ultrasound operators. In general, physicians need further training in ultrasound. Implementation of a general dating ultrasound examination seems to be a relevant goal as most health professionals agree that pregnant women would benefit from this service. An opportunity to further improve maternity care services in Rwanda would involve allowing midwives to become educated in performing basic ultrasound examinations.
